# Epigenetic regulation of the transcriptional activity of genes mediating the neuroprotective activity of ceftriaxone in the brain in a rat model of sporadic Alzheimer disease: the involvement of histone deacetylase 1

**DOI:** 10.18699/vjgb-26-91

**Published:** 2026-10

**Authors:** A.A. Bashirzade, M.A. Tikhonova, V.M. Belichenko, L.A. Fedoseeva, M.D. Belyaev, T.-Y. Renn, H.-M. Chang, N.G. Kolosova, N.D. Belyaev, T.G. Amstislavskaya

**Affiliations:** Scientific Research Institute of Neurosciences and Medicine, Novosibirsk, Russia; Scientific Research Institute of Neurosciences and Medicine, Novosibirsk, Russia; Scientific Research Institute of Neurosciences and Medicine, Novosibirsk, Russia; Scientific Research Institute of Neurosciences and Medicine, Novosibirsk, Russia Institute of Cytology and Genetics of the Siberian Branch of the Russian Academy of Sciences, Novosibirsk, Russia 3; Institute of Cytology and Genetics of the Siberian Branch of the Russian Academy of Sciences, Novosibirsk, Russia; Taipei Medical University, Taipei, Taiwan; Taipei Medical University, Taipei, Taiwan; Institute of Cytology and Genetics of the Siberian Branch of the Russian Academy of Sciences, Novosibirsk, Russia; Scientific Research Institute of Neurosciences and Medicine, Novosibirsk, Russia; Scientific Research Institute of Neurosciences and Medicine, Novosibirsk, Russia

**Keywords:** Alzheimer’s disease, histone deacetylase, ceftriaxone, neuroprotection, amyloid metabolism, BDNF, болезнь Альцгеймера, гистоновая деацетилаза, цефтриаксон, нейропротекция, метаболизм амилоида, BDNF

## Abstract

Histone deacetylases (HDACs) are enzymes that provide transcriptional repression, while HDAC inhibitors are considered to be potential drugs in the treatment of Alzheimer disease (AD). Earlier we found that ceftriaxone (CEF), an antibiotic with neuroprotective activity, augmented mRNA levels of Ide and Mme, encoding enzymes involved in the catabolism of amyloid beta in the amygdala of OXYS rats, a model of sporadic AD. On the other hand, disturbances of neurotrophic factors (BDNF in particular) play an important role in AD pathogenesis. Moreover, HDACs of class I (HDAC1) are involved in the regulation of Bdnf transcriptional activity. The study was aimed to assess the effects of CEF on HDAC1 enrichment at Mme, Ide, and Bdnf gene promoters in the amygdala of OXYS rats and on Bdnf mRNA levels in the amygdala of OXYS and Wistar rats. Chromatin immunoprecipitation and qPCR were utilized for epigenetic analysis and assay of Bdnf mRNA levels, respectively. Neither significant HDAC1 enrichment, nor its alteration under CEF treatment at the Mme or Ide promoters were found, whereas CEF attenuated HDAC1 binding at Bdnf promoter IV. This is a novel mechanism of transcription regulation by CEF in the brain, which may be involved in its neuroprotective activity. In 5-month-old OXYS rats, it seems to play a minor role, as CEF did not change Bdnf transcript 5 (containing exons IV and IX) levels. We were first to demonstrate the transcript-specific regulation of Bdnf transcription by CEF. CEF significantly increased the levels of Bdnf transcript 6 (containing exons VII, VIII, and IX). The results of our study can be taken into account in AD treatment.

## Abstract (ru)

Гистоновые деацетилазы (HDAC) – это ферменты, обеспечивающие подавление транскрипции генов, а ингибиторы HDAC рассматриваются как потенциальные лекарственные средства для лечения болезни Альцгеймера (БА). Ранее мы обнаружили, что цефтриаксон, антибиотик с нейропротекторной активностью, повышает уровни мРНК генов Ide и Mme, кодирующих ферменты, которые участвуют в катаболизме амилоида- бета, в миндалине мозга у крыс линии OXYS, модели спорадической БА. С другой стороны, нарушения нейротрофических факторов (в частности, BDNF) играют важную роль в патогенезе БА. Более того, HDAC класса I (HDAC1) участвуют в регуляции транскрипционной активности Bdnf. Целью данного исследования было оценить влияние цефтриаксона на обогащение HDAC1 в промоторах генов Mme, Ide и Bdnf в миндалине у крыс OXYS и на уровни мРНК Bdnf в миндалине крыс OXYS и Вистар. Для эпигенетического анализа и определения уровней мРНК Bdnf использовали хроматин-иммунопреципитацию и количественную ПЦР, соответственно. Не выявлено значимого обогащения HDAC1 или его изменений под действием цефтриаксона в промоторах Mme и Ide. В то же время цефтриаксон ослаблял связывание HDAC1 с промотором IV Bdnf. Это новый механизм регуляции транскрипции в головном мозге цефтриаксоном, который может быть задействован в его нейропротекторной активности. У 5-месячных крыс OXYS он, по-видимому, играет минорную роль, поскольку цефтриаксон не изменял уровни транскрипта 5 Bdnf, содержащего экзоны IV и IX. Мы также впервые продемонстрировали транскрипт- специфическую регуляцию транскрипции Bdnf с помощью цефтриаксона. Цефтриаксон значительно повысил уровни транскрипта 6 Bdnf, содержащего экзоны VII, VIII и IX. Результаты исследования могут быть учтены при терапии БА.

## Introduction

Abbreviations Aβ, amyloid-beta; AD, Alzheimer’s disease; AICD, APP intracellular domain; APP, amyloid precursor protein; BDNF, Brain-Derived Neurotrophic Factor; BDNF/Bdnf, gene for BDNF; CA, cornu Ammonis of the hippocampus; CEF, ceftriaxone; ChIP, chromatin immunoprecipitation; FP, forward primers; Gapdh, gene encoding glyceraldehyde 3-phosphate dehydrogenase; HDAC, histone deacetylase; Hprt1, gene encoding hypoxanthine phosphoribosyltransferase 1; IDE, Insulin-degrading enzyme; IDE/Ide, gene for IDE; MME, membrane metalloendopeptidase; MME/Mme, gene for MME; NFκB, nuclear factor kappa B; NMDA receptor, N-methyl-D-aspartate receptor; qPCR, quantitative real-time PCR; RP, reverse primers; TSS, Transcription Start Site; tr.var., transcript variant

Alzheimer’s disease (AD) is an incurable multifactorial neurodegenerative disorder characterized by a progressive cognitive decline induced by the accumulation of amyloid beta (Aβ) in the brain, which triggers synaptic plasticity dysfunction, neuronal deficits, and memory loss. Most of the current AD therapies do not stop the disease progression and provide only temporary symptomatic relief. Hence, the development of new treatment strategies that target the key pathogenetic factors in AD development is needed.

Epigenetic factors are considered to make a significant contribution to AD development. Many studies have demonstrated the involvement of different epigenetic mechanisms in the pathogenesis of neurodegeneration in AD (Nikolac Perkovic et al., 2021). Most often, this concerns DNA methylation/ demethylation (Poon et al., 2020), posttranslational modifications of histones (Ramazi et al., 2020), and regulation by various forms of noncoding RNAs (Idda et al., 2018). Acetylation/ deacetylation of histones at lysine is one of the most common and studied mechanisms of chromatin compaction (Shvedunova, Akhtar, 2022). It is regulated by two types of enzymes: histone acetyltransferases and histone deacetylases (HDACs). HDACs are enzymes that remove acetyl groups from lysine residues in the amino terminal tails of histone proteins. The resulting chromatin compaction, in turn, causes transcriptional and translational repression (Sada et al., 2020). HDAC inhibitors are considered potential drugs in AD treatment (Bondarev et al., 2021).

Increasing the expression and/or enzymatic activity of proteins involved in Aβ cleavage has long been considered a promising targeted approach in AD treatment (Zhang et al., 2023). Neprilysin or membrane metalloendopeptidase (MME), being the major amyloid-degrading enzyme in the brain, plays a significant role in AD pathogenesis, in particular, in the late onset sporadic form (Nalivaeva et al., 2020). Regulation of MME expression by HDACs has been revealed in cell cultures. In SH-SY5Y cells, the endopeptidase promoter interacts with HDAC1 and lacks typical histone acetylation marks, such as H4K16 and H4K8. In human HUVEC cells, 15-fold enrichment of MME promoters in HDAC1 has been observed (Belyaev et al., 2009).

Another metallopeptidase, insulinase, also known as insulindegrading enzyme (IDE), together with MME is one of the main enzymes that participate in the extra- and intracellular degradation of Aβ (Kurochkin et al., 2018). Enrichment of the IDE gene promoter in HDACs of class I (but not class III) was significantly reduced in the cell culture of neuron-like SH- SY5Y cells overexpressing APP751 and APP770 (Nalivaeva et al., 2016).

Ceftriaxone (CEF) is a third-generation cephalosporin antibiotic with neuroprotective activity. The drug is extensively applied in preclinical studies on models of neurological disorders. The studies have revealed beneficial effects of CEF in AD-like pathology (Zumkehr et al., 2015; Hefendehl et al., 2016; Tikhonova et al., 2017, 2021). Moreover, longterm treatment with CEF, even over several months, is found to be safe in laboratory rodents (Ratti et al., 2015) and in humans in clinical trials on CEF as a neuroprotective agent in amyotrophic lateral sclerosis (Cudkowicz et al., 2014) or Parkinson’s disease dementia.( National Library of Medicine. To assess the efficacy and safety of ceftriaxone in patients with mild to moderate Parkinson’s disease dementia. ClinicalTrials.gov ID NCT03413384. https://clinicaltrials.gov/ct2/show/ NCT03413384 (Accessed March, 11, 2026)). Suppression of glutamateinduced excitotoxicity was long regarded as the main neuroprotective effect of CEF. However, additional mechanisms of its neuroprotective effects such as an activity targeted at the proteostasis network or pathological aggregation of proteins are suspected (Kumari, Deshmukh, 2021). Indeed, our group has shown that CEF attenuates Aβ load in the brains of mice with Aβ-induced AD-like pathology (Tikhonova et al., 2021). Moreover, CEF changes the transcriptional activity of genes encoding enzymes involved in the catabolism of Aβ. In OXYS rats, which are a model of accelerated senescence and sporadic AD (Stefanova et al., 2014), administration of CEF resulted in the restoration of cognitive and neuronal deficits (Tikhonova et al., 2017) associated with an increase in the mRNA levels of Ide and Mme in the amygdala (Rasero et al., 2017) but not in the hippocampus or frontal cortex (Tikhonova et al., 2018). Amygdala is a brain structure that is closely associated with learning and memory function and is involved in the pathogenesis of AD since the early stages of disease progression. However, the mechanisms of the effect of CEF on the transcriptional activity of endopeptidases remain unknown. Here, we checked whether CEF might influence the expression of genes encoding MME and IDE through the epigenetic regulation of HDAC1 enrichment of the gene promoters.

The search for means enhancing the clearance of intracellular amyloid oligomers using a system of degrading enzymes is not the only direction being developed for AD therapy. There are other approaches, in particular, those related to increasing neuroprotection and neurotrophic function in brain structures. BDNF is the most abundant neurotrophic factor in the central nervous system. It supports the processes of neurogenesis and neuroplasticity and it is involved in learning and memory processes. According to the results of meta-analyses, patients with AD have decreased BDNF levels in the blood and brain (Du et al., 2018; Ng et al., 2019). Moreover, CEF restores the reduced BDNF levels in the striatum of rats in the MPTPinduced model of Parkinson’s disease (Kaur, Prakash, 2017). Earlier we found that alterations in neurotrophic balance indicating increased apoptosis precede the development of ADlike pathology in OXYS rats. Rapid progression of AD-like pathology in OXYS rats is accompanied by the suppression of activity of the neurotrophin system (Rudnitskaya et al., 2017). Hence, we assumed a modulating effect of CEF on the transcriptional activity of Bdnf in the brain of OXYS rats. On 1 National Library of Medicine. To assess the efficacy and safety of ceftriaxone in patients with mild to moderate Parkinson’s disease dementia. ClinicalTrials.gov ID NCT03413384. https://clinicaltrials.gov/ct2/show/ NCT03413384 (Accessed March, 11, 2026). the other hand, previous studies revealed that the modulation of the transcriptional activity of Bdnf was associated with alterations in HDAC1 binding at the Bdnf-IV promoter in the hippocampus of early-life iron-deficient rats (Tran et al., 2015).

Thus, this study was aimed to assess the effect of CEF on the mRNA levels of Bdnf in the amygdala of OXYS and Wistar rats and to evaluate the involvement of enrichment of the gene promoters in HDACs of class I (HDAC1) in the CEF effects on the expression of Mme, Ide, and Bdnf genes in the amygdala of OXYS rats.

## Materials and methods

**Experimental procedures involving animals.** The experiment was performed with 14-week-old male OXYS rats developed at the Federal Research Center Institute of Cytology and Genetics, Siberian Branch of the Russian Academy of Sciences, Novosibirsk, Russia, and male Wistar rats of the control parental strain of the same age. The animals were kept in cages (40 × 60 × 20 cm), five rats in each, with free access to standard chow for laboratory rats and water under standard conditions: a natural light–dark cycle 16 h light : 8 h dark, temperature 18–22 °C, relative humidity 50–60 %. The animals were divided into four experimental groups: OXYS Control, OXYS CEF, Wistar Control, and Wistar CEF. The Control groups were administered saline intraperitoneally (i.p.) while the CEF groups were treated with CEF i.p. at a daily dose of 100 mg/kg. Rats received 36 daily i.p. injections of CEF or saline according to the previously described design in the study of the effect of CEF on the expression of genes related to the system of Aβ metabolism in the brain (Tikhonova et al., 2018). The rationale behind the CEF dosage – 100 mg/(kg·day) – and treatment duration adopted in the current study was based on our previous studies showing neuroprotective effects of CEF in correcting the behavioral and neuronal deficits in OXYS rats (Tikhonova et al., 2017) and the modulating effect of CEF on the expression of Mme and Ide in the amygdala of OXYS rats (Tikhonova et al., 2018). On day 37 the rats were sacrificed by exposure to CO2 and decapitation. Immediately upon sacrifice, the amygdalae were rapidly dissected on ice, frozen in liquid nitrogen, and stored at −70 °C until further analysis.

The study of gene promoter enrichment in HDAC1 was performed in biosamples of the amygdala of rats from the OXYS Control (n = 5) and OXYS CEF (n = 5) groups, as we had not revealed significant effects of CEF treatment on the mRNA levels of Ide and Mme in Wistar rats (Tikhonova et al., 2018). The Bdnf transcriptional activity was assessed by mRNA levels in biosamples of the amygdala in rats of the OXYS Control (n = 8), OXYS CEF (n = 10), Wistar Control (n = 8), and Wistar CEF (n = 10) groups.

**Chromatin immunoprecipitation (ChIP).** The ChIP procedure was performed according to the previously published protocol (Zuccato et al., 2007) with minor modifications. Amygdala tissue was crushed into small pieces in a mortar with liquid nitrogen using a ceramic pestle. Next, the tissue mass was immersed in 1 % formaldehyde solution in PBS to fix DNA and proteins and intensively mixed for 10 min at 4 °C. Fixation was stopped by adding 1.25 M glycine solution. Then the samples were washed three times with cooled PBS solution and immersed in 1 mL of lysis buffer (10 mM Tris-HCl, pH 8.0, 140 mM NaCl, 1 mM EDTA, 1 % Triton X-100, 0.1 % deoxycholate, 1 mM PMSF). The samples were homogenized by passing through a 21G syringe needle. Ultrasonic treatment (sonication) of samples was performed using a Sonopuls mini20 sonicator (Bandelin, Germany) with a power of 20 W in a volume of 1 mL. Sonication mode used: eight times for 30 s with 2-min intervals. The DNA fragments obtained under these conditions were 500–1000 bp long. Then the samples were divided into three unequal parts: 10 % of the volume was allocated for input (i. e. DNA fragments with ligated protein, not treated with immune antibodies) and two equal parts, one for the interaction with specific antibodies against HDAC1 (Catalog # PA1-860, Invitrogen, Thermo Fisher Scientific, United States) and the other for the interaction with control nonimmune antibodies IgG (Catalog # 02- 6102, Invitrogen, Thermo Fisher Scientific, United States). The input was stored at –20 °C until the stage of de-cross-linking the DNA and protein fractions. The ratio of antibody volume to sample volume was 1:500. The samples with antibodies were incubated at 4 °C overnight.

Protein G Sepharose (Catalog # ab193259, Abcam, UK) was incubated with 5 % BSA overnight. BSA-blocked Sepharose was added to the samples immediately after incubation with antibodies and intensively mixed in a shaker for 1 h at 4 °C. Sepharose samples were sequentially washed with solutions containing increasing concentrations of salts and nonionic detergents. The first wash solution consisted of lysis buffer (10 mM Tris HCl, pH 8.0, 140 mM NaCl, 1 mM EDTA, 1 % Triton X-100, 0.1 % deoxycholate, 1 mM PMSF). The second wash solution consisted of 10 mM Tris HCl, pH 8.0, 250 mM LiCl, 0.5 % Triton X-100, 0.5 % sodium deoxycholate, 1 mM EDTA. Sepharose beads were washed with 1 % SDS in 0.1 M NaHCO3.

Bound DNA and protein fractions including inputs were de-cross-linked by adding 5M NaCl and incubating at 65 °C overnight. The samples were then treated with proteinase K in the buffer containing 0.1 M Tris-HCl, pH 8.0, 50 mM EDTA, and 10 % SDS at 50°C for 1 h. DNA was isolated by phenol-chloroform extraction, precipitated with two volumes of 95 % ethanol, and washed twice with 70 % ethanol. The remaining alcohol was evaporated in a thermostat. The precipitate was dissolved in 22 μL of TE buffer (0.01 M Tris + 0.001 M EDTA, pH 8.0). The amount of isolated DNA was measured in a Nanodrop 2000C spectrophotometer (Thermo Fisher Scientific, United States).

To assess the enrichment of the promoter regions of the Mme, Ide, and Bdnf genes, semiquantitative real-time PCR was performed using a CFX96 Touch amplifier (Bio-Rad, United States) with the SYBR Green fluorescent dye. Sequences of specific primers for the promoters of the target genes (Mme, Ide, and Bdnf ) were selected using Primer 3 (NCBI) (Table 1). The specificity of the primers was checked using the Primer- BLAST online software (National Library of Medicine. Primer-BLAST. https://www.ncbi.nlm.nih. gov/tools/primer-blast/ (Accessed October, 20, 2025)). For Bdnf, we studied promoter IV as previous studies revealed the involvement of HDAC1 binding at the Bdnf-IV promoter in the modulation of the transcriptional activity of Bdnf (Tran et al., 2015). Loci that are homologous to primer sequences in the promoter region of the studied genes are shown in Figures 3a, 4a, and 5a.

**Table 1.**
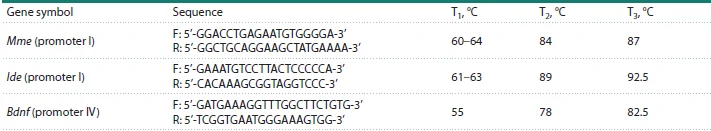
Primers for the promoters of target genes Note. T1: annealing temperature; T2: temperature at fluorescence signal acquisition; T3: product melting temperature.

The obtained amplicons homologous to the fragments of the specified promoters of the target genes in the samples treated with specific antibodies against HDAC1 or control non-immune IgG antibodies and inputs were loaded onto 1 % agarose gel containing ethidium bromide (1 mg/mL). Electrophoresis was performed in a horizontal chamber with 1x TAE buffer at 130 V for 40 min (PowerPac HC, BioRad, United States). The samples were visualized using the Molecular Imager® Gel Doc™ XR System (BioRad, United States). The DNA markers Step100 Long (Catalog # S-8055, Biolabmix, Novosibirsk, Russia) and Step50 plus (Catalog # S-8103, Biolabmix, Novosibirsk, Russia) were used for assessing DNA length.

The enrichment of the promoter regions in HDAC1 was calculated using Percent Input Method (Thermo Fisher Scientific Inc. ChIP Analysis. https://www.thermofisher. com/ru/ru/home/life-science/epigenetics-noncoding-rna-research/ chromatin-remodeling/chromatin-immunoprecipitation-chip/chipanalysis. html (Accessed October, 20, 2025)). The mean Ct was calculated for each antibody type and input (n = 5). The adjusted mean Ct for the input (10 % of total DNA) was calculated as the difference between the mean Ct for the inputs and the binary logarithm of the dilution factor of 10 (log210). For target and non-immune antibodies, the percentage of input was calculated as 100*2ΔCt, where ΔCt is the difference between the adjusted mean Ct for the input and the mean Ct for samples treated with target/non-immune antibodies.

Analysis of mRNA levels of Bdnf transcript variants using qPCR. To analyze the transcriptional activity of the Bdnf gene under the conditions of our experiment, we used two pairs of primers that allowed us to study the expression of two different transcript variants. Despite the final protein product being selfsame, there are 10 validated transcripts of the rat Bdnf gene(National Library of Medicine. NCBI_Gene_Bdnf brain-derived neurotrophic factor [Rattus norvegicus (Norway rat)]. https://www.ncbi.nlm.nih. gov/gene/24225 (Accessed March, 20, 2026)) which are involved in different functions (Baj et al., 2011). Here, we studied the levels of transcript variant 5 (Bdnf-tr.var.5; NM_001270633.1) and transcript variant 6 (Bdnf-tr.var.6; NM_001270634.1). Bdnf-tr.var.5 contains exons IV and IX and is the only transcript specific for exon IV (and its associated promoter region) among all Bdnf transcripts. Bdnf-tr.var.6 contains exons VII, VIII, and IX and is transcribed starting from exon VII – but not the exon IVassociated promoter region; its levels were assessed to evaluate transcript-specific effects of CEF. The primers were mapped onto the gene regions homologous to the transcript variants of interest using the UCSC Santa Cruz Genome Browser(Santa Cruz Genome Browser. 2025. http://genome.ucsc.edu/index.html (Accessed October, 20, 2025)). The specific Bdnf primers for the analysis of mRNA levels of Bdnf-tr.var.5 and Bdnf-tr.var.6 as well as the reference genes Gapdh and Hprt1 were chosen using the Primer-BLAST online software (National Library of Medicine. Primer-BLAST. https://www.ncbi.nlm.nih. gov/tools/primer-blast/ (Accessed October, 20, 2025)).

Extraction of total RNA from fresh-frozen rat amygdala samples was performed using RNeasy Minikit in accordance with the manufacturer’s recommendations (Qiagen, Germany). Residual genomic DNA was removed using DNase I (Promega, United States). The reverse transcriptase reaction was carried out as in (Tikhonova et al., 2018) with 160 units of reverse transcriptase (Biosan, Russia) and 1× RT Buffer (Biosan, Russia). The temperature regime of reverse transcription included three steps: 25 °C for 5 min, 42 °C for 30 min, and 95 °C for 5 min.

The mRNA levels of target and reference genes were measured by qPCR according to a previously published protocol (Belichenko et al., 2023). The reaction was carried out in a LightCycler-480 II thermocycler (Roche, Switzerland) in master mix from a BioMaster HS-qPCR SYBR Blue (2 ×) reagent kit (BIOLABMIX, Novosibirsk, Russia) with forward and reverse specific primers (150 nM each; BIOSSET Ltd., Novosibirsk, Russia; Table 2), and a cDNA template.

**Table 2.**
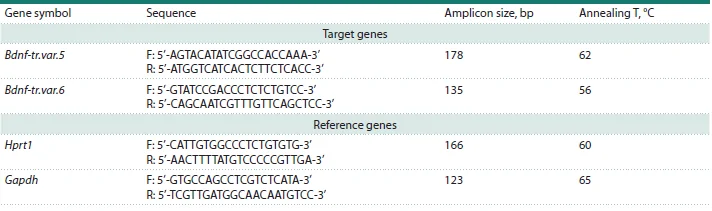
Primers used in qPCR analysis for target and reference genes

All samples were analyzed in triplicate. The relative concentration of a tested cDNA was determined by the standard-curve quantitation method (Ginzinger, 2002) using the LightCycler 480 Software (Release 1.5.1) and calibration curves derived from the dilutions of a standard cDNA. The standard cDNA solution for plotting the calibration curves was prepared by mixing aliquots from each synthesized cDNA sample. The expression value for a target gene was next normalized to the geometric mean mRNA levels of the two reference genes (Hprt1 and Gapdh).

Statistical analysis. All results were presented as mean ± SEM. STATISTICA 10.0 software (StatSoft, Tulsa, OK, United States) was used to perform all the statistical analyses. The normality of the data distribution was determined by the Shapiro– Wilk W test. To compare the enrichment of gene promoters in HDAC1, paired Student’s t test for dependent samples and Student’s t test for independent samples (in Welch’s modification) were used. Statistical evaluation of the Bdnf-tr.var.5 and Bdnf-tr.var.6 transcriptional data was performed by two-way analysis of variance (ANOVA) followed by Bonferroni’s post hoc test. Independent variables for the two-way ANOVA were genotype (Wistar or OXYS strain) and treatment (saline or CEF). The level of significance was defined as p < 0.05 in all experiments reported here. The tendency level was accepted to be 0.05 < p < 0.2.

## Results

**Analysis of the levels of Bdnf transcript variants in the amygdala of OXYS and Wistar rats.** The levels of mRNA species for Bdnf-tr.var.-5 (the only Bdnf transcript containing exon IV) in the amygdala are summarized in Figure 1. According to two-way ANOVA, there was no significant effect of the Treatment factor [F(1, 32) = 1.245, p = 0.274] but there was a tendency toward influence of the interaction between the factors of Treatment and Genotype [F(1, 32) = 2.565, p = 0.120] on the mRNA levels of Bdnf-tr.var.-5 in the amygdala of the rats. The abundance of mRNA species for Bdnf-tr.var.-6 (Bdnf transcript containing exons VII, VIII, and IX) in the amygdala is summarized in Figure 2. According to two-way ANOVA, the Treatment factor significantly influenced the mRNA levels of Bdnf-tr.var.-6 in the amygdala of the rats [F(1, 29) = 4.526, p < 0.05].

**Fig. 1.**
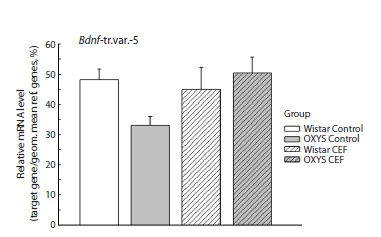
Effects of CEF on Bdnf-tr.var.-5 mRNA levels in the amygdala of OXYS and Wistar rats. Data are presented as a ratio (%) of target transcript mRNA levels to the geometric mean of Gapdh and Hprt1 mRNA levels. The data are expressed as the mean ± SEM of the values

**Fig. 2.**
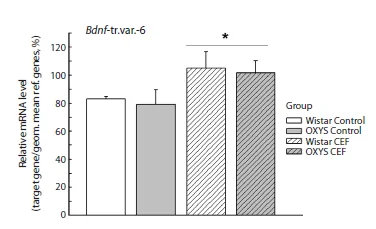
Effects of CEF on Bdnf-tr.var.-6 mRNA levels in the amygdala of OXYS and Wistar rats. The data are presented as a ratio (%) of target transcript mRNA levels to the geometric mean of Gapdh and Hprt1 mRNA levels. The data are expressed as the mean ± SEM of the values obtained in an independent group of animals (n = 5 to 10 per group). Statistically significant differences: * p < 0.05 vs. Control (saline-treated) groups.

**Analysis of the enrichment of gene promoters in HDAC1 in the amygdala of OXYS rats. **In the analysis of the promoter region of Mme gene, the enrichment values for the target antibody against HDAC1 did not differ from the background values for the control IgG antibody according to the t test for independent samples in the Control group (t(8) = 1.35, p > 0.05) or in the group of CEF-treated OXYS rats (t(8) = 0.36, p > 0.05) (Fig. 3). Enrichment of the Ide promoter also did not differ from the background level in either control (t(8) = 0.84, p > 0.05) or CEF-treated group (t(8) = 0.2, p > 0.05) (Fig. 4).

**Fig. 3.**
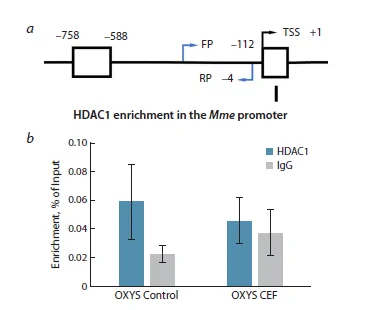
Effects of CEF on rhe enrichment of the Mme promoter in HDAC1 in the amygdala of OXYS rats. (a) Loci that are homologous to primer sequences in the promoter region of the Mme gene are shown. (b) Quantitative results of enrichment in HDAC1 in the promoter region of the gene. Here and in Figures 4 and 5: TSS – Transcription Start Site; FP and RP – Forward and Reverse Primers. The distances (base pairs) from TSS are indicated. Data are expressed as the mean ± SEM of the values obtained in an independent group of animals (n = 5 per group).

**Fig. 4.**
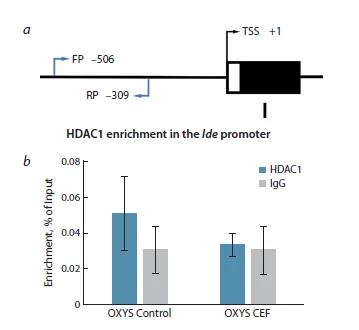
Effects of CEF on enrichment in HDAC1 in the Ide promoter in the amygdala of OXYS rats. (a) The annealing sites for the primers in the promoter region of the Ide gene are shown. (b) Quantitative results of enrichment in HDAC1 in the promoter region of the gene.

We also focused on Bdnf promoter IV. According to previous reports, it is regulated by HDAC1 (Tran et al., 2015) and, accordingly, should be enriched in this protein. Indeed, the enrichment in HDAC1 in the Control group exceeded the background level by a factor of 8 (t(8) = 7.12, p < 0.001). In the CEF-treated group, it was also significantly augmented (t(8) = 2.79, p < 0.05), which motivated us to perform an intergroup comparison for target antibodies against HDAC1. The precipitation rates of the target DNA regions differed significantly between the groups (t(8) = 4.31, p < 0.01). Hence, we infer that CEF affects the degree of HDAC1 enrichment in the Bdnf promoter IV in OXYS rats (Fig. 5).

**Fig. 5.**
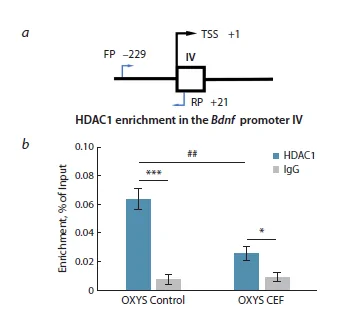
Effects of CEF on enrichment in HDAC1 in the Bdnf promoter IV in the amygdala of OXYS rats. (a) The annealing sites for the primers in the promoter region of the Bdnf exon IV are shown. (b) Quantitative results of enrichment in HDAC1 in the promoter region of the gene. Statistically significant differences: * p < 0.05, *** p < 0.001 vs. corresponding IgG-treated samples; ## p < 0.01 vs. OXYS Control samples treated with antibodies against HDAC1.

## Discussion

It has been reported that CEF significantly affects BDNF expression in the brain. Moreover, CEF exerts a modulating effect on BDNF: it can augment (Kaur, Prakash, 2017; Gao et al., 2024; Cini et al., 2025) or attenuate (Alhaddad et al., 2020; Hammad et al., 2021) its increased levels in different models of neuropathology. Typically, studies of BDNF expression address the levels of the protein-coding exon IX or mature protein. Here, we are the first to demonstrate the transcript-specific regulation of BDNF transcription by CEF. CEF significantly increased the levels of Bdnf-tr.var.-6 (containing exons VII, VIII, and IX) but not of Bdnf-tr.var.-5 (containing exons IV and IX) in the amygdala of OXYS rats. Noteworthy, despite the same final protein product, there are 10 validated transcripts of the rat Bdnf gene(National Library of Medicine. NCBI_Gene_Bdnf brain-derived neurotrophic factor [Rattus norvegicus (Norway rat)]. https://www.ncbi.nlm.nih. gov/gene/24225 (Accessed March, 20, 2026)), which are involved in different functions. For example, the selective activation of dendritic spines of neurons is based on the spatial segregation of different BDNF transcripts. Splice variants 1 and 4 control the architecture of proximal dendrites, whereas variants 2 and 6 regulate distal ones, which leads to differentiated and localized translation into the protein (Baj et al., 2011).

In contrast to the well-studied mechanisms of the antibacterial effect of CEF, its role in neuroprotection remains poorly understood. Although modulating effects of CEF on the expression of many genes in the brain are known, regulating pathways have been studied only for few. One of the known regulatory effects involves NF-κB-mediated activation of promoter of the gene encoding GLT-1 (Lee et al., 2008). However, further studies revealed that this mechanism could be structure-specific: in a rat model of cocaine abuse, CEF strongly upregulated GLT-1 both in the hippocampus and n. accumbens, whereas NF-κB involvement was found in the hippocampus but not in n. accumbens (Niedzielska-Andres et al., 2019). Here, we revealed for the first time a CEF effect on HDAC1 binding in a gene promoter. Indeed, CEF treatment significantly reduced HDAC1 enrichment at the Bdnf promoter IV in OXYS rats. We suggest that this mechanism might be involved in the above-mentioned CEF effect on GLT-1 upregulation in n. accumbens in the rat model of cocaine abuse, as GLT-1 expression was initially suppressed in n. accumbens in rats vulnerable to rewarding effects of cocaine prior the CEF treatment (Niedzielska-Andres et al., 2019). At the same time, this novel epigenetic CEF effect could not be attributed to the CEF-induced upregulation of Mme, Ide, and Bdnf we studied here in OXYS rats.

No HDAC1-mediated effect of CEF on the Mme or Ide promoters was detected. Previously obtained results in a cell culture of rat primary cortical neurons revealed no interaction of HDAC1 with the Mme promoter either (Belyaev et al., 2009). Moreover, in that study, an inverse correlation between HDAC1 enrichment at the MME promoter and MME mRNA levels was found. In cell cultures with low MME mRNA levels (SH-SY5Y, HUVEC), HDAC1 enrichment was the highest. In contrast, in cell cultures with high MME / Mme mRNA levels (NB7, rat cortical neurons), the enrichment was low or null. We did not find reports on HDAC1 enrichment at Ide promoters in rats or rat cells. HDAC1 involvement was also shown in the regulation of IDE in human cell cultures. HDAC1 binding varied in cells overexpressing different splice variants of APP: HDAC1 enrichment was lower in cells overexpressing APP751 and APP770 than in cells injected with APP695 cDNA. IDE mRNA expression was higher in cells with longer APP isoforms, and this observation was consistent with the influence of HDAC1 (Nalivaeva et al., 2016). Noteworthy, untreated 5-month-old OXYS rats did not differ from control Wistar rats in the Mme or Ide mRNA levels in the amygdala (Tikhonova et al., 2018); therefore, the HDAC-mediated gene suppression that might be reverted by HDAC inhibition is unlikely.

In this study, we did not find significant differences between 5-month-old OXYS and Wistar rats in the levels of Bdnf mRNA (Bdnf-tr.var.5 and Bdnf-tr.var.6) in the amygdala. These results are consistent with previous findings about similar Bdnf mRNA levels in the frontal cortex and hippocampus in OXYS and Wistar rats at the age of 14 weeks (Alsallum et al., 2023). At the same time, we revealed the HDAC1 enrichment of promoter IV of the Bdnf gene in 5-month-old OXYS rats, although we observed no significant differences in the levels of Bdnf-tr. var.5 (the only Bdnf transcript containing exon IV in rats) in the amygdala between OXYS and Wistar rats of this age. As a rule, in younger proliferating cells, chromatin is in a less condensed state, which facilitates the access of the cellular transcriptional apparatus. On the contrary, aging cells are characterized by the appearance of densely packed heterochromatin organized into specific structures (Narita et al., 2003). In neurons, which are not prone to proliferation, markers of both active (H3K27Ac) and repressive (H3K27Me3) chromatin may be present, but in aging neurons of the murine hippocampus, a general decrease in acetylation and an increase in deacetylation are observed (Palomer et al., 2016). In the model of accelerated senescence and sporadic AD, enrichment of the BDNF promoter in HDACs seems to be anticipated. Chromatin of glial cells in OXYS rats at the age of 18 months has a high degree of condensation (Stefanova et al., 2015). We are unaware of the degree of enrichment of the Bdnf promoter in HDACs in the early postnatal period, but we could assume it from the state of chromatin in the brain of OXYS rats. For example, the size of the pyramidal neuron nucleus in the CA1 hippocampal area in OXYS rats decreases starting from the age of five months (Stefanova et al., 2014). Hence, we suggest that the activity of HDACs in the early postnatal period is low. With aging, this activity would increase. This is consistent with the present findings on the enrichment of the Bdnf promoter IV in HDAC1 in the amygdala of OXYS rats at the age of five months, when morphological changes in the neuron nuclei appear. Although this regulatory mechanism of gene transcription arises in 5-month-old OXYS rats, it seems to play a minor role at this age as no significant differences were found in the Bdnf-tr. var.5 levels in the amygdala between OXYS and Wistar rats or under the CEF treatment, which significantly reduced HDAC1 binding to the Bdnf promoter IV but did not change Bdnf-tr. var.5 levels in OXYS rats.

## Conclusion

The study of the mechanisms of chromatin remodeling through histone acetylation/deacetylation is of great interest for cognitive and behavioral neuroscience research. Moreover, dysregulation of histone acetylation pathways has been found in the hippocampus and frontal cortex of AD patients (Schueller et al., 2020), while HDAC inhibitors are considered potential drugs in the treatment of AD (Bondarev et al., 2021). Here, we disclosed a novel mechanism of transcription regulation by CEF in the brain related to the inhibition of the HDAC1 enrichment of a gene promoter, which might underlie its neuroprotective activity. However, in 5-month-old OXYS rats, a rat model of sporadic AD, it seems to play a minor role at this age, as CEF treatment significantly reduced HDAC1 binding to Bdnf promoter IV but did not change Bdnf-tr.var.5 (the only Bdnf transcript containing exon IV in rats) levels in the amygdala in OXYS rats. We are also the first to demonstrate the transcript-specific regulation of BDNF transcription by CEF. CEF significantly increased the levels of Bdnf-tr.var.-6 (containing exons VII, VIII, and IX) but not of Bdnf-tr.var.-5 in the amygdala of OXYS rats. The results of the study can be taken into account and used in AD treatment and the correction of cognitive deficits in neurodegenerative diseases.

## Conflict of interest

The authors declare no conflict of interest.
